# A Case in Practice

**Published:** 1870-11

**Authors:** H. Scott

**Affiliations:** Lancaster, O.


### A CASE IN PRACTICE.

BY H. SCOTT.

On the 26th day of September, Mrs. D— called with her husband to have a tooth extracted. She was probably about thirty-five years old, of sanguine-nervous temperament, and seemed to be delicate. A few days previously she called on her family physician to have a tooth taken out, as the distance to Lancaster was eight miles. The doctor advised her to go to the dentist, but she insisted, and he made the attempt. It was the first molar tooth on the left side and very firm. I do not know what instrument he used, but the crown gave way near the bifurcation, leaving the roots a little below the margins of the gums, and the pulp, which protruded, with its membrane intact.

In this condition she came to my office, after four or five days of suffering and as many sleepless nights. She was, of course, greatly exhausted, and her naturally sensitive nervous system was at the same time drawn up to a high state of tension. I had no doubt of being able to grasp the roots and remove them, and made preparations to do so, the nerve pulp being, as I believed, too sensitive to justify an attempt to break it down. I succeeded in securing a safe hold by inserting the points of my forceps between the roots, but found on applying force that I should have a great deal of resistance to overcome. The tooth did not start with my first effort, and before making the second I found my patient jerking and sighing in an ominous way; so much so as to cause me to desist from any further efforts. There were in fact manifest indications of convulsions. We removed her to the lounge, where she rapidly passed into a cataleptic state, from which she did not recover for more than three hours. She became entirely unconscious, and some of the symptoms assumed a rather alarming charactor. The face was pallid, the lips was bluish from the accumulation of carbon in the blood, induced by the difficulty of inflating the lungs, and the pulse at the wrists ceased. These urgent symptoms were presently relieved, however, by the application of carbonate ammonia to the nostrils, which induced deep and full inspirations, and consequent oxidation of the blood. But still the catalepsia continued till the afternoon was well worn away; nor was she able after return to recall any event that had transpired subsequent to taking her seat on the operating chair.

I was afraid of her nerves, and decided not to attempt to a further effort to extract the roots or to excise or break down the nerve, and so I contented myself with sprinkling the pulp with dry arsenic, which I covered with soft cotton. The pulp suppurated, and she is herself again.

I am unable to account for the shock to her nerves in any other way than by reference to their unwonted tension at the moment, for I caused her little or no pain. I am quite sure I did not touch the pulp in the least.

Lancaster, O., Oct. 25, 1870.

